# Distinctive effects of CD34- and CD133-specific antibody-coated stents on re-endothelialization and in-stent restenosis at the early phase of vascular injury

**DOI:** 10.1093/rb/rbv007

**Published:** 2015-06-01

**Authors:** Xue Wu, Tieying Yin, Jie Tian, Chaojun Tang, Junli Huang, Yinping Zhao, Xiaojuan Zhang, Xiaoyan Deng, Yubo Fan, Donghong Yu, Guixue Wang

**Affiliations:** ^1^Key Laboratory of Biorheological Science and Technology (Chongqing University), Ministry of Education, Chongqing Engineering Laboratory in Vascular Implants, Bioengineering College, Chongqing University, No. 174 Shazhengjie, Shapingba, Chongqing 400044, China, ^2^School of Biological Science and Medical Engineering, Beihang University, No. 37 XueYuan Road, HaiDian District, Beijing 100191, China and ^3^Department of Biotechnology, Chemistry, and Environmental Engineering, Aalborg University, Fredrik Bajers Vej 7, Building H, 9220, Aalborg, Denmark

**Keywords:** stent, CD133, CD34, endothelial progenitor cells, re-endothelialization

## Abstract

It is not clear what effects of CD34- and CD133-specific antibody-coated stents have on re-endothelialization and in-stent restenosis (ISR) at the early phase of vascular injury. This study aims at determining the capabilities of different coatings on stents (e.g. gelatin, anti-CD133 and anti-CD34 antibodies) to promote adhesion and proliferation of endothelial progenitor cells (EPCs). The *in vitro* study revealed that the adhesion force enabled the EPCs coated on glass slides to withstand flow-induced shear stress, so that allowing for the growth of the cells on the slides for 48 h. The *in vivo* experiment using a rabbit model in which the coated stents with different substrates were implanted showed that anti-CD34 and anti-CD133 antibody-coated stents markedly reduced the intima area and restenosis than bare mental stents (BMS) and gelatin-coated stents. Compared with the anti-CD34 antibody-coated stents, the time of cells adhesion was longer and earlier present in the anti-CD133 antibody-coated stents and anti-CD133 antibody-coated stents have superiority in re-endothelialization and inhibition of ISR. In conclusion, this study demonstrated that anti-CD133 antibody as a stent coating for capturing EPCs is better than anti-CD34 antibody in promoting endothelialization and reducing ISR.

## Introduction

Percutaneous coronary intervention is an important treatment for occlusive coronary artery disease. Nevertheless, restenosis still remains a major problem of this therapy [1]. The drug-eluting stent (DES) has dramatically reduced the incidence of in-stent restenosis (ISR) [2, 3], but it has been suggested that it could impair the healing process of the injured arterial wall [4]. Moreover, the eluted drug from the stent may lead to delayed re-endothelialization so that it cannot prevent ISR completely [5]. Pathologically, there are two noteworthy interfaces in the vessel segment with stent implantation. The stent interface contacting with blood is the one where platelet aggregation and thrombogenesis may occur, the stent interface contacting with the vessel wall is the other where the proliferation of smooth muscle cells takes place. Pre-endothelialization is regarded as an effective way to suppress ISR [6, 7] by accelerating the re-endothelialization of the injured vessel wall so that the two interfaces aforementioned may not further deteriorate pathologically.

Endothelial progenitor cells (EPCs) are population of specialized adult stem cells circulating in peripheral blood that can anchor at target tissues differentiating and forming new blood vessels. Recent studies showed that EPCs had been identified as a key factor of re-endothelialization [7–9] either by direct incorporation into vessel walls or by secreting a variety of angiogenic growth factors [10]. The early establishment of a functional endothelial layer after vascular injury has been proven to assist the prevention of neointimal proliferation and thrombus formation [11, 12]. Therefore, constructing a biofunctional multilayer on biomaterial surface to capture EPCs might be able to generate a new endothelial layer on the surface. This novel concept has led to the invention of the EPC-capture stents, which use immobilized antibodies targeting at EPCs surface antigens and are considered to be a safe and effective way to reduce complications of stenting, including thrombosis and ISR. Antibody-coated stent could capture EPCs in circulating and adhere them to the stent, as a result accelerating stent endothelialization which depending on the adhesion and differentiation of the captured EPCs.

EPCs are characterized by the expression of CD133, CD34 and the vascular endothelial growth factor receptor-2 (VEGFR2, Flk-1) [13, 14]. In previous studies [15, 16], several substrates including gelatin, VEGFR2, anti-CD34 or anti-CD133 antibody were investigated in terms of the adherent strength of the EPCs on the stent surface. The results showed that the adherent forces of the EPCs on the stent surface coated with anti-CD133 were higher than those coated with anti-VEGFR2 and anti-CD34. In addition, it has been demonstrated that to some extent, shear stress could promote the proliferation and NO secretion of EPCs. The EPCs revealed stronger adhesion on the substrate of anti-CD133 antibody than the other two substrates.

However, these studies did not address the question of which antibodies are suitable to capture EPCs *in vivo*. In this study, assay of *in vitro* tubule formation by EPCs was performed on different antibodies coated on collagen I surfaces. Then, an *in vivo* study using the rabbit abdominal aorta model was carried out to examine the efficiency of two different antibody-coatings on stents in promoting re-endothelialization and suppressing stenosis after implantation.

## Materials and Methods

### EPCs isolation and characterization

Animal housing and surgical procedures conform to the Guide for the Chinese Animal Care and Use Committee standards, which conform to the Guide for the Care and Use of Laboratory Animals published by the US National Institutes of Health (NIH Publication No. 85-23, revised 1996). All animal procedures were also performed in accordance with protocols approved by the Animal Ethics Committee of Chongqing University. NIH guidelines (or for non-US residents similar national regulations) for the care and use of laboratory animals (NIH Publication No. 85-23 Rev. 1985) have been observed.

New Zealand white rabbits (1.5–2.0 kg) were purchased from the Chongqing Medical University (Chongqing, China). EPCs were isolated from bone marrow and cultured afterward on the basis of protocols as described in previous studies [17, 18]. Briefly, bone marrow isolated from rabbit was diluted by endothelial cell growth medium-2 (EGM-2) with 1:1 ratio, and mononuclear cells were separated by density gradient centrifugation with 1.077 g/ml Percoll solution for 20 min at 400 × g. Cells were cultured separately for 3 weeks in EGM-2. Medium was refreshed every 3 days. The non-adherent cells were reseeded in gelatin-coated six-well plates. After 7 days of culturing, non-adherent cells were washed and removed by phosphatic buffer solution (PBS) and adherent cells were continuously cultured in 25-cm^2^ culture flasks and six-well plates. First passage cells were used for further analysis. Phenotypic analysis of the cells was performed. After 7 days of the culture, the expression of several specific antigens of EPCs, VEGFR2/fetal liver kinase-1 (FLK-1) and anti-CD34 and anti-CD133 were analyzed by immunofluorescence and immunocytochemistry. Non-specific antigens CD14, CD29 and CD45 were used as the negative control. All antibodies were purchased from Santa Cruz Biotechnology, Santa Cruz, CA. According to the phenotype and function of cells, cells are concluded for late EPCs.

### In *vit*ro *tubule formation*

Collagen I was placed in 200-µl well in 24-well culture plates. The plates were then incubated at 37°C for 30 min to allow the collagen to solidify. The collagen was overlaid with 500 µl of EGM-2 containing 10^4^ cells per well. The media of some wells were coated with anti-CD34 or anti-CD133 antibody (1%) before seeding cells. Then they were further incubated at 37°C for 4 h [19, 20]. Tube formation was monitored by a phase-contrast microscopy (Olympus, Japan). Average tubule length was obtained from three randomly selected fields per well.

### Coating of stents

The BMS (316 L stainless steel stents, 1.6 mm in diameter, 17 mm in length, Beijing Amsino Medical Devices Co. Ltd., China) were washed separately with distilled water, 75% ethanol, acid pickle, acetone and deionized water by an ultrasonic cleaner. Gelatin (4 µg/ml), gelatin mixed with anti-CD34 antibody and gelatin mixed with anti-CD133 antibody were used as the coating solution. The solutions were sprayed onto the surface of BMS by the way of ultrasonic atomization, stents were freeze-dried at −60°C for 24 h and then expanded with corresponding balloon and confirmed by scanning electron microscopy (SEM).

### Capability of capturing EPCs *in vitro*

To evaluate EPC-capturing ability of gelatin stents, anti-CD34 antibody stents and anti-CD133 antibody stents, we used EPCs in two different circumstances, static condition and dynamic condition. EPCs density used in the experiments was 15 × 10^4 ^cells/ml. Under static condition, coated stents were, respectively, placed in six-well culture plates containing 15 × 10^4 ^cells per well. After 4 h, the cells adhered on the surface of the stents were eluted and counted. Three independent experiments were performed for each treatment for statistical analysis. Under dynamic condition, an extracorporeal circulation system [21] was used to simulate the transfer process *in vitro* (Fig. 5C). Coated stents were, respectively, mounted on a balloon, transported to a gel silica flex tubing, expanded and perfused at various shear rates (2.5, 5 and 10 dynes/cm^2^) in the flow system for 4 h to simulate the planting experiments *in vivo*. Then stents incubated at 37°C for 4 h under static conditions. The cells that adhered on the surface of the stents were eluted and counted. Three independent experiments were performed for each treatment for statistical analysis.

### Stents implantation

The Guide for Chinese Animal Care and Use Committee standards was followed for the Animal housing and surgical procedures were followed to conform to the Guide for the Chinese Animal Care and Use Committee Standards. Male New Zealand white rabbits (1.8–2.5 kg) were anesthetized with an intravenous injection (30 mg/kg) of sodium pentobarbital (30 g/l). The stents were introduced through the femoral artery using a 0.014-inch Steerable guide wire and were placed in the abdominal aorta under fluoroscopic guidance. The stent was deployed by inflating the balloon to 10 atmospheres pressure for 60 s; the balloon was then deflated and was withdrawn. To ensure the correct location and no damage of the vessels, angiograms were obtained immediately after stent implantation. The animals were randomly assigned before the start to receive a bare metal stent, a gelatin-coated stent, an anti-CD34 antibody or anti-CD133 antibody-coated stent.

### SEM examination and histomorphometric analysis

All the animals were monitored daily for stent-related complications, and the stents were harvested 1, 4 and 12 weeks, respectively, after the implantation (*n* = 3, at each time point of each group). Experimental animals were anesthetized with sodium pentobarbital (30 g/l) firstly, then were euthanized by cardiac perfusion with 50 ml of PBS. Vessels implanted stents were dissected from the sacrificed animals. A part of stents samples were fixed in 4% paraformaldehyde and then embedded in light-cured resin through toluidine blue stain for pathology organization analysis. The others were fixed in 0.25% glutaraldehyde for 12–16 h for SEM and morphologic analysis of intimal surface. Morphometric analysis was performed with a computerized digital image analysis system by double-blinded study. Neointima was identified by the observation the changes of the slice thickness and proliferation and migration of smooth muscle cells under optical microscope. Histomorphometric analysis and calculation of each section included lumen area (mm^2^), neointimal area (mm^2^), the neointimal thickness (mm) and percentage of stenosis by Image Tool (version 2.0; UTHSCSA).

### Statistical analysis

The data shown were mean values of at least three independent experiments and expressed as mean ± standard error of the mean. Statistical comparisons for different groups were performed using either the Student’s *t*-test or one-way analysis of variance. Statistical significance was set as *P* < 0.05, *P* < 0.01 being highly significant.

## Results

### Characterization and identification of EPCs

**Fig. 1.**
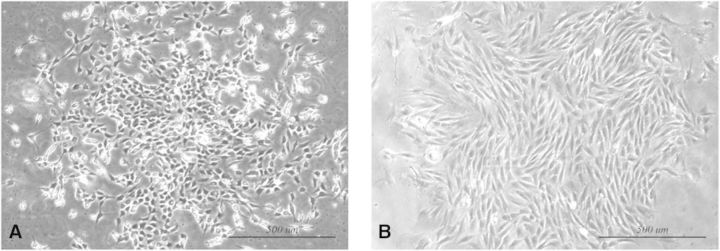
Characterization of EPCs. (**A**) EPCs were isolated from bone marrow for 4 days. (**B**) Secondary colony formation of EPCs after seeding 7 days. Scale bars = 500 m

**Fig. 2.**
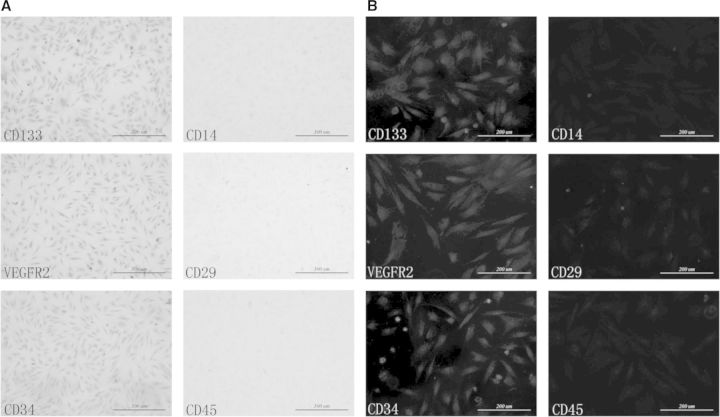
Identification of EPCs by immunocytochemistry and immunofluorescence. (**A**) Immunocytochemistry, scale bars = 500 µm. (**B**) Immunofluorescence, scale bars = 200 µm

After 4 days of culturing, spindle cells with typical EPCs morphology began to form colonies as shown in Figure 1A. Consistent with the functional capacity of a progenitor cell, single cell by limiting dilution formed secondary coloning after 7 days in culture, being illustrated in Figure 1B. The results of immunofluorescence and immunocytochemistry demonstrated that bone marrow-derived EPCs that we isolated were CD133^+^ VEGFR2^+^CD34^+^CD14^−^ CD29^−^ CD45^−^ (Fig. 2).

### *In vitro* tube formation

**Fig. 3.**
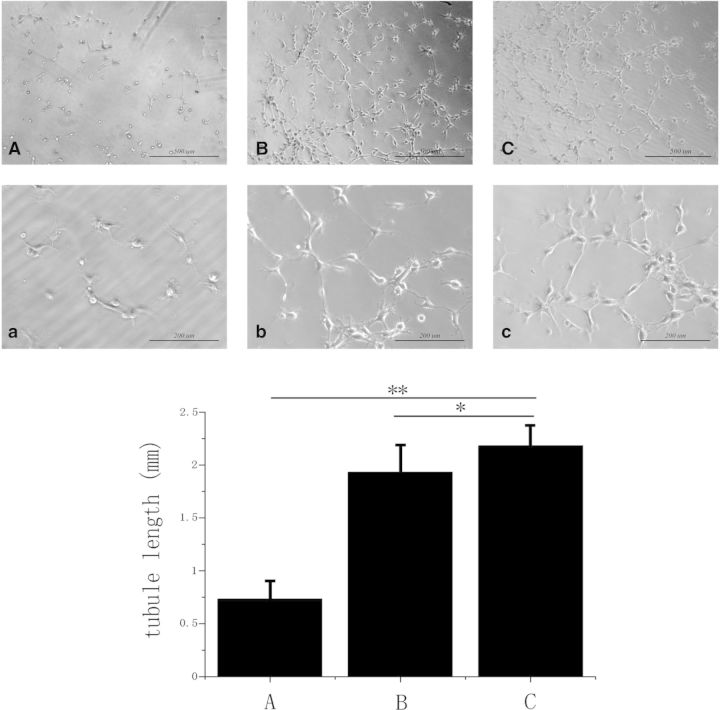
The *in vitro* tube formation assay of EPCs. (**A**) Without coating, scale bars = 500 µm; (**a)**: without coating, scale bars = 200 µm. (**B**) Anti-CD34 antibody coating, scale bars = 500 µm; (**b**): anti-CD34 antibody coating, scale bars = 200 µm. (**C**) Anti-CD133 antibody coating, scale bars = 500 µm; (**c**): anti-CD133 antibody, scale bars = 200 µm (*P < 0.05; **P < 0.01)

The EPCs without coating in small round shapes were close to isolated cells but still had a number of connecting branches between two cells as presented in Figure 3A and a. EPCs with coated anti-CD34 antibody can be seen in Figure 3B and b, and anti-CD133 as found in Figure 3C and c led to the development of capillary tubes and formation of cellular networks. Result of statistics showed that both anti-CD34 and anti-CD133 significantly increased the tube-like formation of EPCs, and the tubule length on anti-CD133 coating was greatly larger than that on anti-CD34 coating (Fig. 3).

### Coating on stents

**Fig. 4.**
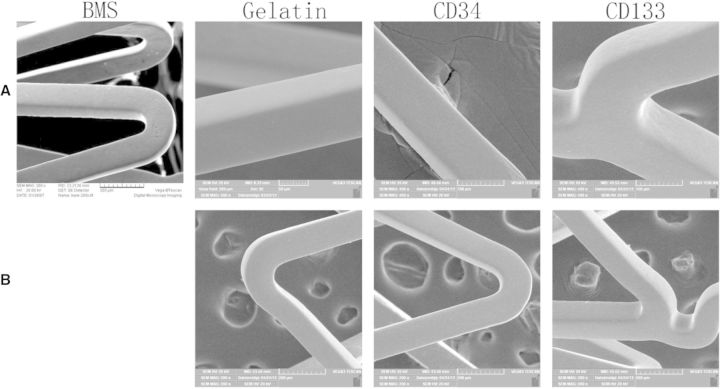
Preparation of coating stents. (**A**) SEM observed the surface of stents before expanded. (**B**) SEM observed the surface of stents after expanded. BMS, bare metal stent; glutin, gelatin-coated stent; CD34, anti-CD34 antibody-coated stent; CD133, anti-CD133 antibody-coated stent

As shown in Figure 4A, SEM study proved a rough surface of the BMS, whereas gelatin formed a uniformed layer over the entire protein-coated stent. The surface of antibody-coated stents was covered with a smooth membrane without aggregation (Fig. 4A). Each stent was coated with about 1.0 ml of protein solution. After vacuum freeze-drying and expanded, the coatings remained intact (Fig. 4B).

**Fig. 5.**
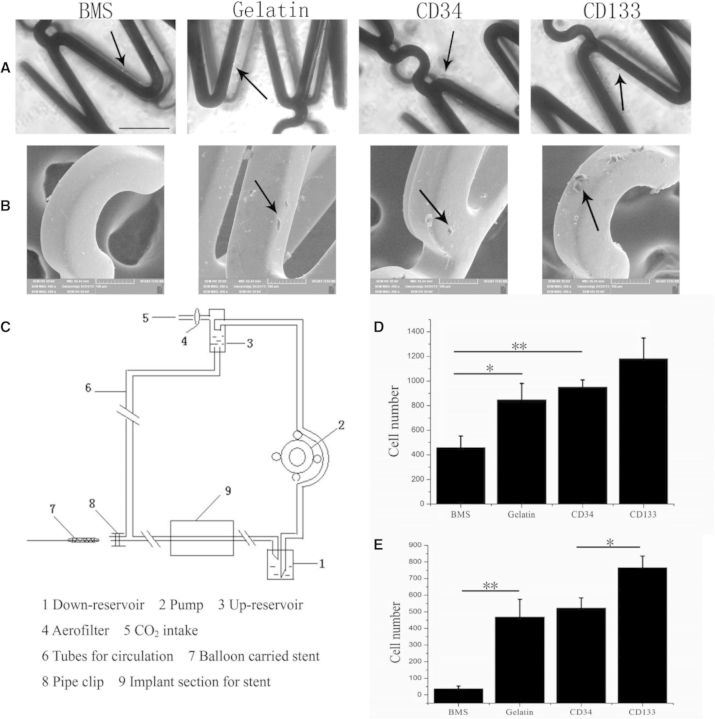
The capability to capture EPCs *in vitro*. (**A**) Phase-contrast microscopy observed the surface of stents after static culturing, scale bars = 500 µm. (**B**) SEM observed the surface of stents after culturing with shear force. (**C**) The extracorporeal circulation system. (**D**) Quantification of number of captured cells in the static condition. (**E**) Quantification of number of captured cells with shearforce (shear rate 2.5 dynes/cm^2^). The arrow showed cells adhesion on the surface of stents

### Capability of capturing EPCs *in vitro*

After static culturing, there were some EPCs adhered on the surface of all kinds of stents (Fig. 5A). Result of statistics showed that, antibody-coated stents achieved remarkable fraction of cell coverage on the surface than gelatin-coated stents or BMS. Although the cell adhesion rate of anti-CD133 antibody-coated stents are higher than anti-CD34 antibody-coated stents but no significant differences between both of them (Fig. 5D).

Figure 5C exhibited the equipment for testing the capability of capture EPCs of stents with shear force. Through flow culturing, all stents have no EPCs adhered at shear rates 5 dynes/cm^2^ and 10 dynes/cm^2^. At shear rate 2.5 dynes/cm^2^, no cells could be observed on the surface of bare metal stents but cell debris, but all the coated stents have cells adhered which were spread (Fig. 5B). Result of statistics shows that, the cells adhered on the coating stents were dramatically more than those on the bare metal ones, and the capture ability of anti-CD133-coated stents was sufficiently better than anti-CD34-coated ones (Fig. 5E).

### Stent implantation

**Fig. 6.**
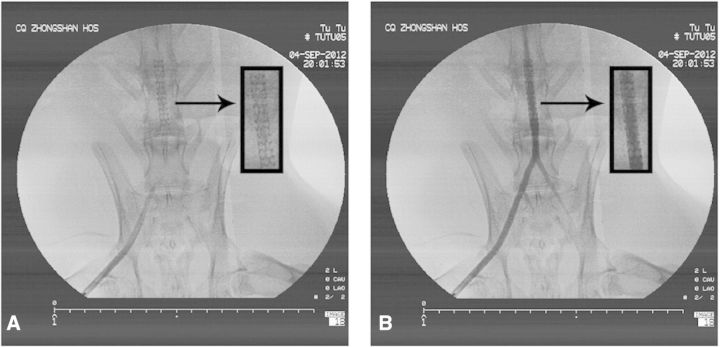
Angiograms obtained immediately after stents implantation. (**A**) All deployment procedures were successful. (**B**) All vessels implanted stents were competent. The arrows show the position of stents implantation

Angiograms obtained immediately after stent implantation showed that all deployment procedures were successful (Fig. 6A), and all stented vessels were competent (Fig. 6B). Reexamination angiograms at different periods revealed that there was no shift of the stents, aneurysm or thrombus in the vessels.

### Stent morphology

**Fig. 7.**
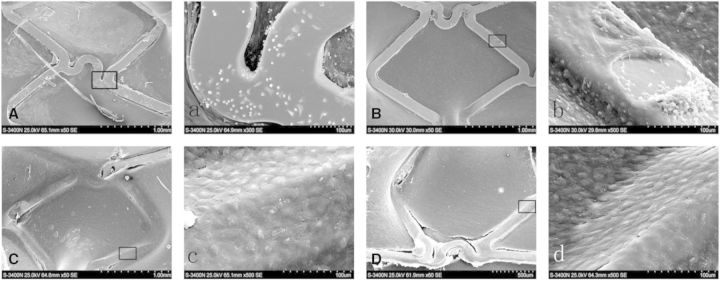
SEM of stents demonstrated the rate of endothelial coverage after 1 week. (**A**, **a**): bare metal stent; (**B**, **b**): gelatin-coated stent; (**C**, **c**): anti-CD34 antibody-coated stent and (**D**, **d**): anti-CD133 antibody-coated stent

One week after the implantation, SEM showed that large numbers of platelets and red cells covering the non-endothelialized areas of BMS (Fig. 7A and a) and the surfaces of gelatin coating stents (Fig. 7B and b) were covered by a discontinuous membrane, which covered <50% of the stent surface, and there were also some adhered blood cells, whereas antibody-coated stents were almost completely covered with cells after stent implantation (Fig. 7C and c; 7D and d), and some cells on the surface of anti-CD133-coated stent were spindle (Fig. 7D and d). Because of the shape of endothelial cells would change from cobblestone to spindle in the presence of shear stress, the results were revealed that the time of cells adhesion was longer and earlier present in the ani-CD133 antibody-coated stents compared with the CD34 ones.

**Fig. 8.**
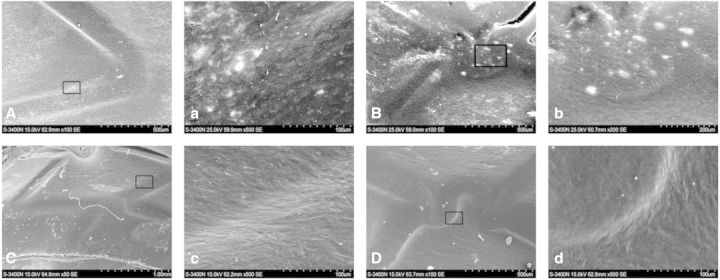
SEM of stents demonstrated the rate of endothelial coverage after 4 weeks. (**A**, **a**): bare metal stent; (**B**, **b**): gelatin-coated stent; (**C**, **c**): anti-CD34-coated stent and (**D**, **d**): anti-CD133-coated stent

Bare metal stents (Fig. 8A and a) and gelatin-coated stents (Fig. 8B and b) were covered by new intima with some adhered corps and fissured at 4 weeks. The surfaces of antibody-coated stents were almost the same as their surfaces at 1 week (Fig. 8C and c; 8D and d). The cells on antibody-coated stent surfaces became longer and morphologically more close to the normal endothelial layer.

**Fig. 9.**
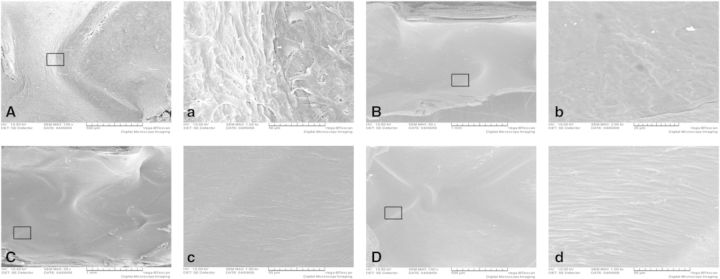
SEM of stents demonstrated the rate of endothelial coverage after 12 weeks. (**A, a**): bare metal stent; (**B, b**): gelatin-coated stent; (**C**, **c**): anti-CD34-coated stent and (**D**, **d**): anti-CD133-coated stent

After 12 weeks, a monolayer of cells was not found, and fibrous structures was found on the surface of bare metal stents (Fig. 9A and a) and gelatin-coated ones (Fig. 9B and b). Surface of anti-CD34 antibody (Fig. 9C and c) -coated stents were more glossy than the sample of 4 weeks, whereas the anti-CD133 antibody-coated stents (Fig. 9D and d) remained the same as the sample of 4 weeks indicating that anti-CD133 antibody-coated ones accomplished the intimal agglutination 4 weeks after the implantation and the stented vessels re-established the steady state of normal vessels.

### Neointima formation after stent implantation

**Fig. 10.**
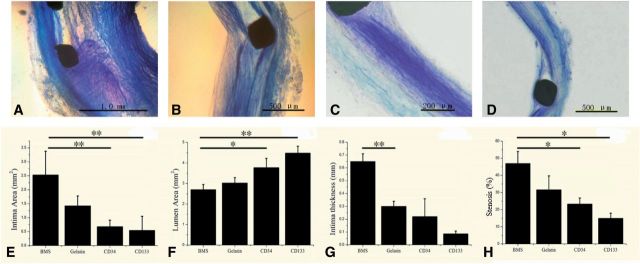
Results of the tissue slice. Representative sections of arteries at the sites of stent struts demonstrating neointimal growth at 12 weeks and histomorphometric measurements. (**A**) Bare metal stent; (**B**) gelatin-coated stent; (**C**) anti-CD34-coated stent; (**D**) anti-CD133-coated stent; (**E**) statistical result of intima area for four kinds of stents; (**F**) statistical result of lumen area for four kinds of stents; (**G**) statistical result of intima thickness for four kinds of stents and (**H**) statistical result of stenosis for four kinds of stents

After antibody-coated stents implantation for 12 weeks, the stent segments were removed for the preparation of hard tissue slicing. After toluidine blue staining, different structure of the vascular wall was observed. Figure 10 exhibited representative arterial cross-sections from different treatment groups. The quantitative morphometric analysis for each of the stent groups is summarized in the second row. After 12 weeks, anti-CD34 (Fig. 10C) and anti-CD133 (Fig. 10D) antibody-coated stents markedly reduced the intima area (Fig. 10E) and increased the lumen area (Fig. 10F) than bare metal stents (Fig. 10A) and gelatin-coated stents (Fig. 10B). The intima thickness (Fig. 10G) and stenosis (Fig. 10H) were also significantly reduced compared with those for bare metal stent. Although there was no statistical significance between two kinds of antibody-coated stents, statistical result showed that anti-CD133 antibody-coated stents have superiority in inhibition of ISR (*P* = 0.7).

## Discussion

Because of its potent efficacy to inhibit restenosis that has been the limitation of BMS, DES with anti-proliferative drug has changed the treatment pattern of interventional cardiology. However, DES also revealed disadvantages such as delayed healing of the stented arterial segment. Delayed arterial healing, characterized by persistent fibrin deposition and poor endothelialization, has been shown to correlate with late DES thrombosis, especially for ‘off-label’ use. The lack of functional endothelium with subsequent inflammatory cells infiltration into the injured artery and hypersensitivity reaction to polymers on DES can cause activation of thrombotic cascade resulting in stent thrombosis [22]. To overcome these limitations, a number of attempts have been made to develop new coronary stents. A ‘pro-healing’ approach has been developed to capture circulating EPCs to achieve rapid recovery of natural endothelium of the stent surface. Once an endothelial layer forms as a functional barrier, it can prevent several pathologic consequences, such as blood clot formation and infiltration of inflammatory cells or proliferation of vascular smooth muscle cells.

EPCs are precursors of endothelial cells. Growing evidences suggested that bone marrow-derived EPCs circulating in the blood may play an important role in the formation of new blood vessels and contribute to vascular homeostasis for adults [23]. CD34, VEGFR2 and CD133 are important antigens and specific to identify EPCs [13, 14]. EPCs have the ability to migrate to sites of vascular injury and aid the regeneration of damaged and dysfunctional endothelium [24]. Clinically, the safety of EPCs-capture stent has been proven in numerous clinical trials with low incidence of late stent thrombosis. As an innovative strategy, EPCs capture stents have been developed, i.e. Genous™ stent (OrbusNeich, Fort Lauderdale, FL), which is coated with murine monoclonal anti-human CD34 antibodies. This stent has been evaluated through several single-arm clinical studies and shown to be safe and feasible for the treatment of coronary artery disease [12, 25, 26]. Many studies showed that biofunctional multilayers composed of anti-CD34 coated on stents can significantly improve the blood compatibility and the endothelialization of the medical devices [27, 28]. But very limited studies were performed on anti-CD133 antibody as a coating to capturing EPC. The study by Sedaghat *et al.* [29] showed that *in vitro,* the stent coating with anti-human CD133-antibodies could successfully achieve effective binding of CD133-positive cells. However, *in vivo*, no difference in re-endothelialization or neointimal formation was evident with the use of anti-CD133 antibody stents when compared with BMS. In their study, no comparison was made between the anti-CD133-coated stent and anti-CD34 antibody-coated one. The exploration of this field would be of great importance for the treatment of coronary artery disease.

In this study, we selected different methods to combining anti-CD133 antibody with stent and compared four kinds of stent (BMS, gelatin-coated stent, anti-CD34 antibody-coated stent and anti-CD133 antibody-coated stent), respectively. In the *In vivo* studies [29, 30], re-endothelialization had been reported to occur within 4 weeks in the rabbit external iliac vessels after stent implantation. After 12 weeks, anti-CD133-coated stents showed reduced neointimal area and stenosis when compared with bare metal stents, gelatin-coated and anti-CD34 antibody-coated stents. The restenosis of BMS can be dramatically decreased by using antibody-coated BMS, especially anti-CD133-coated BMS. Our results both *in vitro* and *in vivo* suggested that anti-CD133 antibody-coated stents could be a new therapeutic strategy to reduce in-stent neointimal formation because no evidence of either systemic or local adverse effects was observed in the rabbits tested. Yu *et al.* [31] found that, it could enhance re-endothelialization of injured vessels via a direct action on EPC differentiation, whereas the mRNA expression of CD133 was strikingly decreased. It is possible that CD133 play a role in EPC differentiation. To validate the therapeutic strategy via rapid re-endothelialization to promote vascular healing, more studies are needed, among them, EPC capture stents would be a more feasible approach.

It is worth to mention that this study has its limitations. For instance, the cells covering the stents *in vivo* should have been identified by immunostains and the selection of vector system could be further improved.

Nevertheless, this study has, for the first time, demonstrated the safety and feasibility of implanting intra-coronary stents coated with anti-CD133 antibody in an animal model and proved that anti-CD133 antibody as a stent coating for capturing EPCs is better than anti-CD34 antibody both *in vitro* and *in vivo*. The results showed the effectiveness of anti-CD133 antibody-coated stents for promoting endothelialization and reducing ISR. This approach can accelerate tissue regeneration without provoking excessive neointima. The material contained anti-CD133 antibody may be potent on vascular graft surface modifier.
